# Preliminary Evaluation of SGLT2 Inhibitors in Human Bladder Cancer T24 Cells

**DOI:** 10.3390/biomedicines14092037

**Published:** 2026-09-10

**Authors:** Martyna Szachniewicz, Michał Baran, Maciej Gagat, Marta Hałas-Wiśniewska

**Affiliations:** 1 Students’ Research Group of Cell Biology and Ultrastructure at the Department of Histology and Embryology, Faculty of Medicine, Collegium Medicum in Bydgoszcz, Nicolaus Copernicus University in Toruń, 85-092 Bydgoszcz, Poland; martynaszachniewicz@wp.pl (M.S.);; 2 Department of Histology and Embryology, Faculty of Medicine, Collegium Medicum in Bydgoszcz, Nicolaus Copernicus University in Toruń, 85-092 Bydgoszcz, Poland; mgagat@cm.umk.pl; 3 Department of Morphological and Physiological Sciences, Faculty of Medicine, Collegium Medicum, Mazovian Academy in Płock, 09-402 Płock, Poland

**Keywords:** bladder cancer, SGLT2 inhibitors, T24 cell line, in vitro

## Abstract

**Background/Objectives:** Sodium–glucose cotransporter 2 (SGLT2) inhibitors have recently gained attention for their potential anticancer properties. However, their effects in bladder cancer remain poorly understood. In this preliminary study, we assessed the effects of canagliflozin, dapagliflozin, and empagliflozin on metabolic activity, cell cycle distributions, migratory behavior, and SGLT2 expression in the human bladder cancer cell line T24. **Methods:** Cellular metabolic activity, cell cycle distribution, apoptosis and necrosis, migration, and SGLT2 protein expression were assessed using 3-(4,5-Dimethylthiazol-2-yl)-2,5-diphenyltetrazolium bromide (MTT) assay, flow cytometry, wound-healing and single-cell migration assays, Western blotting, and fluorescence microscopy. Bioinformatic analyses of *SLC5A2* were additionally performed. **Results:** All three compounds reduced MTT-derived metabolic activity in a concentration-dependent manner and induced G0/G1 cell-cycle accumulation without increasing apoptosis or necrosis after 24 h. Flozins also impaired collective wound closure and altered single-cell migration parameters, although replicate-level analysis showed no significant differences between individual treatment conditions and control after multiple-comparison correction. SGLT2 protein was detected in T24 cells and decreased following treatment, with compound-dependent differences in magnitude. **Conclusions:** Flozins affect metabolic activity, cell-cycle progression, wound closure, and migratory behavior in T24 cells. However, these findings do not establish SGLT2-specific mechanisms and were obtained at concentrations exceeding typical clinical plasma exposure. Further target-validation and mechanistic studies are required.

## 1. Introduction

Bladder cancer (BC) is the ninth most common cancer worldwide, with over 614,000 new cases and 220,000 deaths reported in 2022. It is the sixth most common cancer in men and the 17th in women [1]. Despite being less common in women, female patients are often diagnosed at a more advanced stage, resulting in worse survival rates [2]. Tobacco smoking is the leading risk factor for BC, accounting for approximately 50% of cases [3]. Other significant risk factors include occupational exposure to aromatic amines, polycyclic aromatic hydrocarbons, and chlorinated hydrocarbons, as well as ionizing radiation [4]. Urothelial carcinoma is the most common BC subtype, comprising over 90% of cases [5]. Approximately 75% of BC patients are diagnosed with non-muscle-invasive bladder cancer (NMIBC; stages Ta–T1), whereas muscle-invasive bladder cancer (MIBC; stages T2–T4) is associated with a poorer prognosis and higher cancer-specific mortality [6]. Systemic treatment options for advanced or metastatic urothelial carcinoma have expanded substantially with the introduction of immune checkpoint inhibitors and antibody–drug conjugates, and recent network meta-analyses indicate that combination regimens incorporating both modalities achieve the greatest improvements in survival outcomes compared with chemotherapy alone [7]. Despite these advances, acquired chemoresistance remains a major obstacle to durable disease control, with recent work characterizing distinct molecular adaptation strategies across chemoresistant bladder cancer cell lines exposed to cisplatin, vinblastine, and gemcitabine [8]. In parallel, recent patient-derived organoid–immune co-culture studies have highlighted the complexity of the urothelial tumor microenvironment and identified heterogeneous mechanisms associated with resistance to immunotherapy, further emphasizing the need to explore complementary biological vulnerabilities in bladder cancer [9].

Sodium–glucose cotransporter 2 (SGLT2) is a transmembrane protein and a member of the solute carrier (SLC) family, encoded by the *SLC5A2* gene. SGLT2 is selectively expressed on the luminal membrane of the S1 and S2 segments of the renal proximal tubule [10,11]. There, it is responsible for reabsorbing over 90% of filtered glucose in the kidney, operating in a 1:1 ratio with sodium [12,13]. Because malignant cells exhibit increased glucose demand and metabolic reprogramming, glucose transporters have emerged as potential therapeutic targets in cancer. Beyond its physiological role in renal glucose homeostasis, SGLT2 expression has also been observed in various cancer types, including breast, prostate, pancreatic, lung, and liver cancer [14,15,16,17].

SGLT2 inhibitors, also known as flozins, are a relatively new class of antihyperglycemic agents used in the treatment of type 2 diabetes mellitus (T2DM). They lower plasma glucose by preventing the reabsorption of filtered glucose from the tubular lumen, making this an insulin-independent mechanism [12]. They are also administered in heart failure and chronic kidney disease regardless of diabetes status, due to their cardioprotective and nephroprotective effects [18,19]. They reduce the risk of worsening heart failure, cardiovascular death, acute kidney injury, and progression of kidney disease [20,21]. To date, four SGLT2 inhibitors have been approved by the Food and Drug Administration (FDA) for use in adults: empagliflozin, dapagliflozin, canagliflozin, and ertugliflozin [22]. Emerging evidence indicates that flozins may also have anticancer properties, with numerous studies reporting antiproliferative effects, inhibition of glycolytic metabolism, induction of apoptosis, reduced tumor growth, and suppressed angiogenesis across various cancer types, including lung, liver, breast, prostate, and pancreatic cancers [14,15,16,17,23,24,25]. These observations have increased interest in targeting tumor metabolism using drugs originally developed for metabolic disorders.

Despite growing evidence supporting the anticancer activity of SGLT2 inhibitors in several malignancies, their biological effects in bladder cancer remain incompletely understood. To date, only limited experimental data are available regarding the biological effects of SGLT2 inhibition in bladder cancer models. Therefore, additional experimental studies are needed to determine whether these compounds exert comparable biological effects in bladder cancer. The T24 cell line was selected as a well-established experimental model of high-grade urothelial carcinoma that is widely used in studies investigating bladder cancer biology and therapeutic responses. Accordingly, the aim of this preliminary in vitro study was to evaluate the effects of canagliflozin, dapagliflozin, and empagliflozin on metabolic activity, cell cycle progression, migratory behavior, and SGLT2 expression in the human bladder cancer T24 cell line. This study was designed as an initial biological characterization of the cellular response to flozin treatment in bladder cancer cells, providing a foundation for future mechanistic and target-validation studies.

## 2. Materials and Methods

### 2.1. Cell Culture and Treatment

The human bladder cancer T24 cell line (ATCC HTB-4; American Type Culture Collection, Manassas, VA, USA) was provided by the Department of Histology and Embryology, Nicolaus Copernicus University in Toruń, Collegium Medicum in Bydgoszcz. Cells were cultured under recommended conditions (5% CO_2_, 37 °C, 95% humidity) in appropriate culture vessels (25 cm^2^ and 75 cm^2^ flasks; 6-, 12- and 24-well plates) depending on experimental design. For optimal growth, McCoy’s 5A (Lonza, Basel, Switzerland) was supplemented with 1% penicillin–streptomycin (P/S, Corning, New York, NY, USA) and 10% fetal bovine serum (FBS, Corning). To ensure reproducibility, cells were used up to passage 6 and regularly monitored for Mycoplasma contamination (4′,6-diamidino-2-phenylindole (DAPI) staining, Merck KGaA, Darmstadt, Germany).

Cells were treated with SGLT2 inhibitors at concentrations selected based on the 3-(4,5-Dimethylthiazol-2-yl)-2,5-diphenyltetrazolium bromide (MTT, Merck KGaA, Darmstadt, Germany) assay results. Empagliflozin (E), dapagliflozin (D) and canagliflozin (C) (TargetMol, Wellesley Hills, MA, USA) were applied at 10 µM and 20 µM. The concentrations selected for subsequent experiments were based on the MTT assay and were chosen to induce measurable biological responses while maintaining sufficient cell viability for downstream analyses. Flozin stock solutions were prepared in dimethyl sulfoxide (DMSO, Avantor, Gliwice, Poland) and diluted in culture medium immediately before use. The final DMSO concentration did not exceed 0.1% in any experimental group. Control (CTRL) cells were cultured under identical conditions without flozin treatment.

### 2.2. MTT Assay

Cellular metabolic activity was assessed using the MTT assay. The tested compounds were used at concentrations of 10, 15, 20, 25, and 30 µM. After 24 h of incubation with flozins, cells were washed with PBS and incubated with MTT solution (Merck KGaA, Darmstadt, Germany) for 2 h. Subsequently, the formed formazan crystals were dissolved in DMSO (37 °C, 10 min, Avantor, Gliwice, Poland). Absorbance was measured at 570 nm using a spectrophotometer (Spectra Academy, K-MAC, Anyang, South Korea). The results were normalized to the CTRL group, which was set as 100%. Data are presented as mean ± standard deviation (SD). Concentration–response curves were analyzed by nonlinear regression using a normalized inhibitor-response model with a variable slope (GraphPad Prism, version 6, GraphPad Software, San Diego, CA, USA). Half-maximal inhibitory concentration (IC_50_) values and corresponding 95% confidence intervals (CI) were estimated from the fitted curves. Data were obtained from three independent biological experiments.

### 2.3. Apoptosis and Cell Cycle Analysis

Cell death was evaluated using Annexin V/propidium iodide (AV/PI) double staining. A commercial kit containing Annexin V Alexa Fluor 647, PI, and annexin-binding buffer (ABB) (Elabscience^®^, Houston, TX, USA) was used according to the manufacturer’s instructions. After 24 h of incubation with selected flozin concentrations (10 and 20 µM), T24 cells were trypsinized, collected and centrifuged (8 min, 300× *g*). Cell pellets were resuspended in ABB buffer, followed by the addition of 1 µL of AV and 1 µL of PI (20 min, RT, in the dark). Stained cells were analyzed using a Guava 6HT-2L flow cytometer (Merck KGaA, Darmstadt, Germany), and data were processed using FlowJo software (v10, FlowJo LLC, Ashland, OR, USA).

Cell cycle distribution was determined by flow cytometric analysis of DNA content using 7-aminoactinomycin D (7-AAD). Cells from all experimental groups, both CTRL and flozins-treated T24 cells, were fixed in cold methanol (−20 °C, for 24 h), washed with cold PBS (3 × 5 min) and stained with 7-AAD (20 min, RT, in the dark; Thermo Fisher Scientific, Waltham, MA, USA). DNA content was analyzed using a Guava 6HT-2L flow cytometer (Merck KGaA), and the distribution of cells in the G0/G1, S, and G2/M phases of the cell cycle was evaluated using FlowJo software (v10, FlowJo LLC).

### 2.4. Western Blot

The semiquantitative analysis of SGLT2 protein expression was performed using a standard Western blot protocol. CTRL and flozins-treated cells were trypsinized, washed with cold PBS and cell pellets were stored at −80 °C until use. Next, pellets were lysed in IP buffer (Tris/NaCl/NP-40, protease and phosphatase inhibitors). Lysates were cleared by centrifugation, mixed with loading buffer, and denatured at 95 °C for 5 min. Proteins were separated on 10–20% Tris-Glycine gels (Novex™ WedgeWell™ Thermo Fisher Scientific, Waltham, MA, USA) and transferred to nitrocellulose membranes (iBlot^®^2, Invitrogen Life Technologies, Carlsbad, CA, USA).

Membranes were blocked with 2.5% milk in Phosphate-Buffered Saline with Tween-20 (PBST), incubated overnight at 4 °C with primary antibodies including SGLT2 polyclonal (Proteintech, Chicago, IL, USA) and glyceraldehyde 3-phosphate dehydrogenase (GAPDH) as a loading control, followed by incubation with appropriate secondary antibodies. Signals were detected using chemiluminescence (SuperSignal™ West Pico PLUS, Thermo Fisher Scientific, Waltham, MA, USA) and imaged with the ChemiDoc MP system (Bio-Rad, Hercules, CA, USA). Band intensities were quantified using ImageJ software (version 1.54p, National Institutes of Health (NIH), Bethesda, MD, USA).

### 2.5. Wound Healing Assay and Open-Field Migration Assay

For the collective cell migration wound healing (WH) assay, T24 cells were seeded in 24-well plates and cultured until approximately 100% confluence to allow the formation of a uniform monolayer. Next, a linear scratch was created in the center of each well using a sterile pipette tip. For the single-cell migration (the open-field cell migration; OF assay), cells were seeded at a density of 0.1 × 10^5^ cells per well. In both experiments, cells were washed with PBS and subsequently treated with selected flozins at concentrations of 10 and 20 µM. The plates were transferred to an inverted motorized microscope (Carl Zeiss AG, Oberkochen, Germany) equipped with a live-cell imaging incubation system (PeCon GmbH, Erbach, Germany). The imaging setup included an EC Plan-Neofluar 10×/0.30 Ph1 air objective, an Axiocam 503 mono camera, and ZEN 2 software (Zeiss). Images were acquired at 10 min intervals for 24 h from predefined positions in each well. The wound area (WH) and additional cell migration parameters (OF: cumulative and Euclidean distance; velocity; and directionality) were quantified using ImageJ software (version 1.54p, NIH, Bethesda, MD, USA) and Chemotaxis and Migration Tool (version 2.0, Ibidi GmbH, Gräfelfing, Germany), respectively. Directionality was calculated as the ratio of Euclidean distance to accumulated distance, with higher values indicating more directionality in persistent migration. For the open-field (OF) assay, 20–30 cells per condition were tracked within each of three independent biological experiments. Individual trajectories were used to illustrate the distribution and heterogeneity of single-cell motility parameters. However, cells originating from the same experiment were not considered independent biological replicates. For inferential statistical analysis, individual cell measurements were averaged within each independent experiment for each experimental condition, resulting in three biological replicate-level values per condition (n = 3).

### 2.6. Fluorescent Labeling of Proteins

Fluorescence staining was performed to visualize SGLT2 and F-actin. CTRL and flozin-treated T24 cells grown on slides in 12-well plates were fixed with 4% paraformaldehyde (PFA, 20 min, RT, Thermo Fisher Scientific, Waltham, MA, USA). Subsequently, cells were permeabilized with 0.25% Triton X-100 (10 min, RT) and blocked with 1% bovine serum albumin (BSA) (1 h, RT, Sigma-Aldrich, St. Louis, MO, USA). Cells were incubated with primary antibodies against SGLT2 (mouse monoclonal, 1:100, 1 h, RT; Santa Cruz Biotechnology, Dallas, TX, USA), followed by incubation with secondary antibody (Alexa Fluor 564, 1:200, 1 h, RT, in the dark, Life Technologies (Thermo Fisher Scientific), Carlsbad, CA, USA). After washing with PBS (3 × 5 min, RT), F-actin was stained using Alexa Fluor 488 phalloidin (1:40, 20 min, RT, in the dark, Invitrogen, Life Technologies, Carlsbad, CA, USA). Finally, the cell nuclei were counterstained with DAPI (1:1000; 10 min, RT, in the dark; Sigma-Aldrich, St. Louis, MO, USA). Slides were washed with PBS and mounted with Aqua Poly/Mount (Polysciences Inc., Warrington, PA, USA). The prepared slides were evaluated using a Nikon Eclipse E800 fluorescence microscope and NIS-Elements 4.0 software (Nikon, Tokyo, Japan) and a C1 confocal microscope equipped with a 60× oil immersion objective and Nikon EZ-C1 software v3.80 (Nikon Corporation, Tokyo, Japan).

Confocal fluorescence images were acquired using identical acquisition settings for all samples. Fluorescence intensity linescan analysis was performed in Fiji/ImageJ (NIH, Bethesda, MD, USA) by drawing representative lines across individual cells and measuring fluorescence intensity profiles for SGLT2 and F-actin channels.

### 2.7. Statistical Analysis

All experiments were performed in at least three independent biological replicates. Data are presented as mean ± SD. The normality of data distribution was assessed using the Shapiro–Wilk test.

For analyses involving two independent variables, two-way analysis of variance (ANOVA) followed by Dunnett’s multiple-comparisons test was used. For comparisons among multiple groups with one independent variable, ordinary one-way ANOVA followed by Tukey’s multiple-comparisons test or Dunnett’s multiple-comparisons test was applied, depending on the experimental comparison. For data that did not meet the assumptions of parametric analysis, the Kruskal–Wallis test followed by Dunn’s multiple-comparisons test was used. For comparisons of normalized values against a theoretical value of 100%, the one-sample Wilcoxon test was applied. A *p*-value < 0.05 was considered statistically significant. For the open-field migration assay, individual tracked cells were not considered independent biological replicates. Cell-level data were therefore used descriptively to illustrate the distribution and heterogeneity of migration parameters. For inferential analysis, measurements from individual cells were averaged within each independent biological experiment and experimental condition, yielding n = 3 biological replicates per condition. Replicate-level data were analyzed using the Friedman test for matched observations, followed, where appropriate, by Dunn’s multiple-comparisons test comparing individual treatment conditions with CTRL. Statistical analyses were performed using GraphPad Prism (version 6 GraphPad Software, San Diego, CA, USA).

### 2.8. Analysis of SGLT2 Based on Available Databases

To provide additional biological context for the experimental findings, publicly available databases were used to investigate the expression, clinical relevance, molecular interactions, and functional associations of *SLC5A2* (SGLT2).

Gene expression and overall survival analyses were performed using the UALCAN platform (http://ualcan.path.uab.edu, accessed on 15 July 2025), which provides access to The Cancer Genome Atlas (TCGA) level 3 RNA-seq and clinical datasets for cancer transcriptome analysis [26,27]. Genomic alterations of *SLC5A2* were evaluated using the cBioPortal platform (https://www.cbioportal.org, accessed on 15 July 2025) based on the TCGA Bladder Urothelial Carcinoma (PanCancer Atlas) dataset [28,29,30]. Potential microRNAs targeting *SLC5A2* were predicted using the miRDB database (http://mirdb.org, accessed on 15 July 2025) [31,32].

To investigate the biological context of *SLC5A2*, gene interaction networks were generated using GeneMANIA (https://genemania.org, accessed on 15 July 2025) with default parameters for Homo sapiens [33]. Protein–protein interaction analysis was performed using the STRING database (https://string-db.org, accessed on 15 July 2025) with a minimum interaction score of 0.4 [34]. Functional enrichment analysis of genes associated with the *SLC5A2* interaction network was subsequently conducted using the Enrichr platform (https://maayanlab.cloud/Enrichr, accessed on 15 July 2025) based on the Reactome (https://reactome.org, accessed on 15 July 2025) and KEGG (https://www.kegg.jp, accessed on 15 July 2025) pathway databases [35,36,37,38,39,40,41].

## 3. Results

### 3.1. Expression of SGLT2 in T24 Bladder Cancer Cells

**Figure 1:**
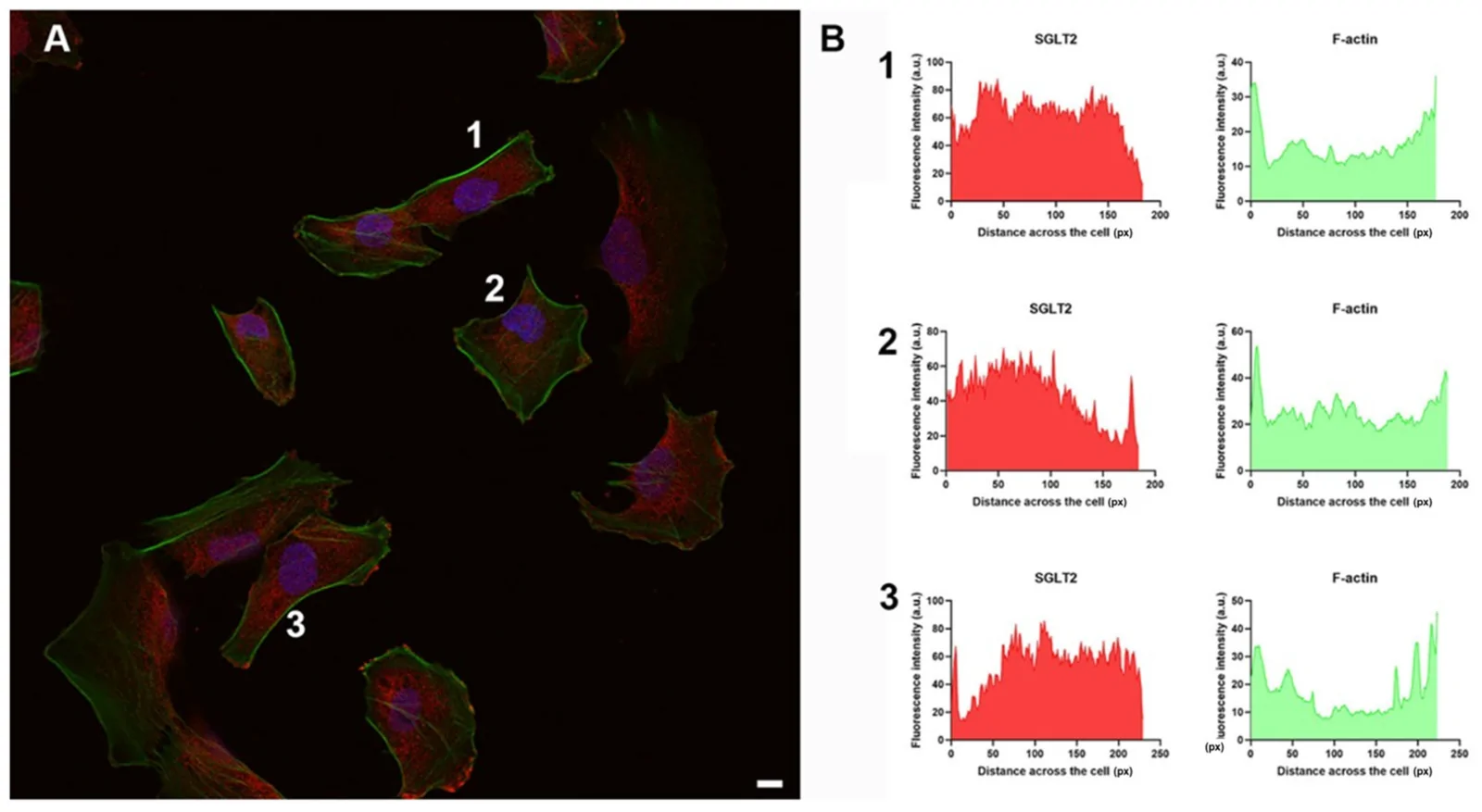
Confocal fluorescence imaging and linescan analysis of SGLT2 expression in T24 bladder cancer cells. (**A**) Representative confocal fluorescence images of T24 bladder cancer cells stained for SGLT2 (red), F-actin (green), and cell nuclei (blue). Numbers indicate representative cells selected for fluorescence intensity analysis. Scale bar = 50 µm. (**B**) Representative fluorescence intensity profiles generated along lines drawn across individual cells. Red plots represent SGLT2 fluorescence intensity, while green plots correspond to F-actin fluorescence intensity. SLGT2—sodium-glucose cotransporter.

Confocal fluorescence microscopy demonstrated detectable SGLT2 expression in T24 bladder cancer cells. The fluorescence signal was distributed throughout the cytoplasm, with locally increased intensity in peripheral cellular regions adjacent to cortical F-actin structures (Figure 1A). F-actin staining clearly outlined cell morphology and cytoskeletal organization, allowing visualization of the spatial relationship between SGLT2 immunofluorescence and the cell periphery. Representative fluorescence intensity line-scan analysis confirmed a broad intracellular distribution of the SGLT2 signal, with local increases in fluorescence intensity observed in selected peripheral regions corresponding to cortical actin organization (Figure 1A,B).

### 3.2. Flozins Reduce Metabolic Activity and Alter Cell Cycle Distribution in T24 Cells Without Inducing Apoptosis at 24 h

**Figure 2:**
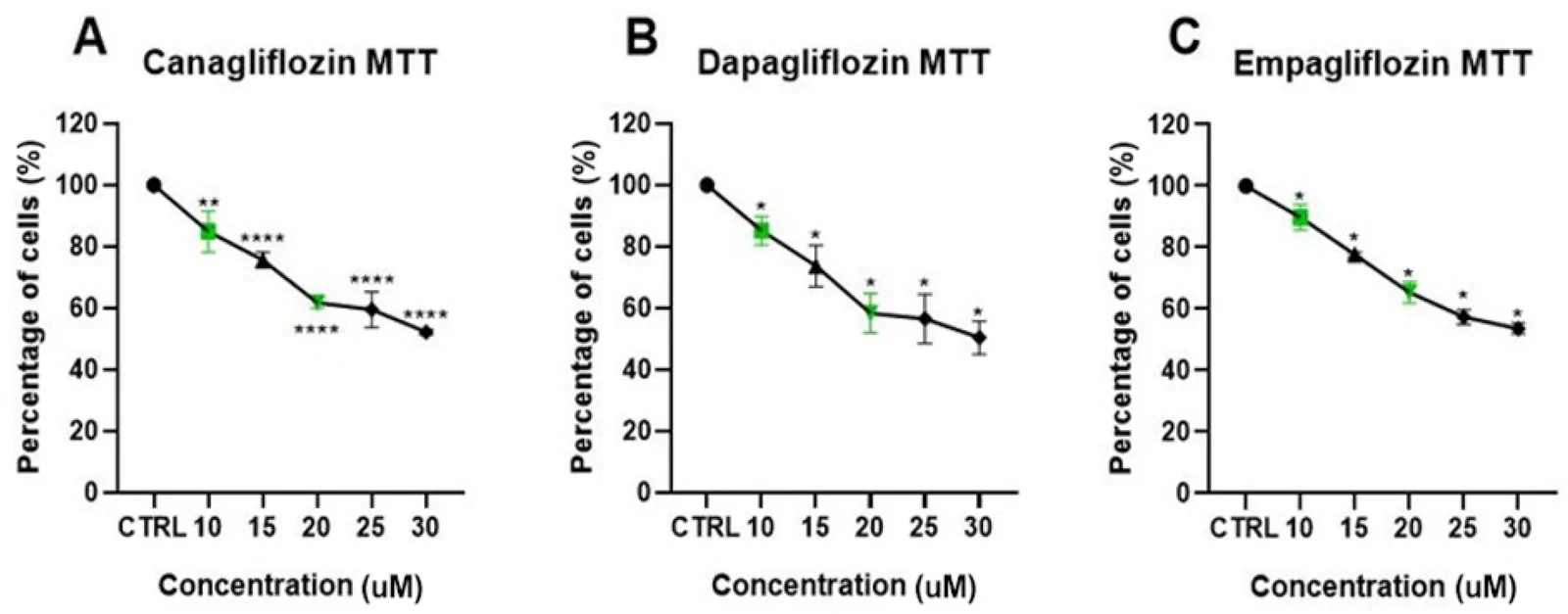
Concentration-dependent effects of SGLT2 inhibitors on MTT-derived metabolic activity in T24 cells. T24 cells were treated with increasing concentrations (10–30 µM) of canagliflozin (**A**), dapagliflozin (**B**), and empagliflozin (**C**) for 24 h. Cellular metabolic activity was assessed by MTT assay and expressed as percentage of control (CTRL = 100%). Nonlinear concentration–response analysis yielded estimated IC_50_ values of 31.30 µM (95% CI: 28.38–35.96 µM) for canagliflozin, 28.89 µM (95% CI: 25.63–34.75 µM) for dapagliflozin and 30.78 µM (95% CI: 28.88–33.34 µM) for empagliflozin. IC_50_ values were estimated using a normalized inhibitor-response model with a variable slope. Data are presented as mean ± SD from 3 independent experiments (n =3). Statistically significant differences are marked as * *p* < 0.05, ** *p* < 0.01 and **** *p* < 0.0001 (One-sample Wilcoxon test). SGLT2—sodium-glucose cotransporter 2, MTT—3-(4,5-Dimethylthiazol-2-yl)-2,5-diphenyltetrazolium bromide, CTRL—control cells. IC_50_—half-maximal inhibitory concentration.

The effect of SGLT2 inhibitors on T24 bladder cancer metabolic activity was evaluated using the MTT assay (Figure 2). Treatment with all three compounds resulted in a concentration-dependent reduction in MTT-derived metabolic activity compared with CTRL. Canagliflozin significantly decreased metabolic activity starting at 10 µM (84.92 ± 6.63%), with further reductions observed at 15 µM (76.65 ± 2.61%), 20 µM (61.76 ± 1.75%), 25 µM (59.52 ± 5.77%), and 30 µM (52.28 ± 0.89%) (Figure 2A). Dapagliflozin also significantly reduced metabolic activity, with effects evident at 10 µM (85.26 ± 4.72%) and progressively decreasing to 73.75 ± 6.74%, 58.36 ± 6.39%, 56.56 ± 7.92%, and 50.41 ± 5.39% at 15, 20, 25, and 30 µM, respectively (Figure 2B). Similarly, empagliflozin treatment led to a significant reduction in metabolic activity beginning at 10 µM (89.87 ± 4.15%), reaching 77.78 ± 0.73%, 65.41 ± 3.45%, 57.41 ± 2.32%, and 53.68 ± 1.78% at 15, 20, 25, and 30 µM, respectively (Figure 2C). Overall, all three compounds produced a comparable, concentration-dependent reduction in MTT-derived metabolic activity across the tested range. Nonlinear concentration–response analysis yielded estimated IC_50_ values of 31.30 µM (95% CI: 28.38–35.96 µM) for canagliflozin, 28.89 µM (95% CI: 25.63–34.75 µM) for dapagliflozin and 30.78 µM (95% CI: 28.88–33.34 µM) for empagliflozin. The overlapping confidence intervals suggest broadly comparable estimated effects among the three compounds within the tested concentration range.

**Figure 3:**
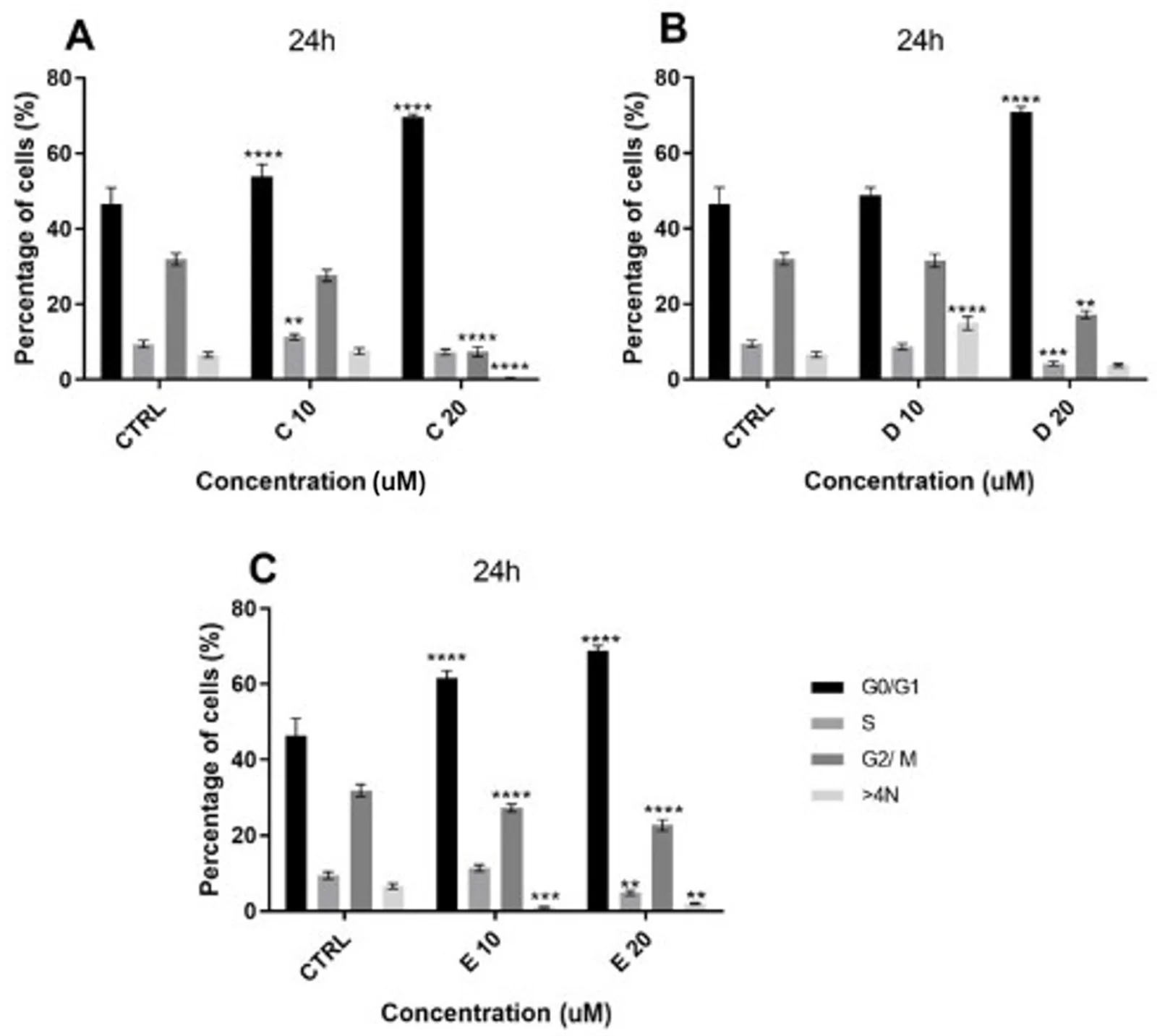
Alterations in cell cycle progression of T24 cells following SGLT2 inhibitor treatment. Cell cycle analysis of T24 cells following treatment with 10 and 20 µM of canagliflozin (**A**), dapagliflozin (**B**), and empagliflozin (**C**) for 24 h. The percentages of cells in G0/G1, S, G2/M, and >4N phases are shown. Data represent mean ± SD from 3 independent experiments (n = 3). Statistically significant differences are indicated as ** *p* < 0.01, *** *p* < 0.001, **** *p* < 0.0001 (2-way ANOVA with Dunnett’s multiple comparisons test). CTRL—control cells, C—canagliflozin, D—dapagliflozin, E—empagliflozin, SGLT2—sodium-glucose cotransporter 2.

Cell cycle analysis demonstrated that SGLT2 inhibitors significantly affected the distribution of T24 cells across different phases (Figure 3). Canagliflozin induced a significant accumulation of cells in the G0/G1 phase at 20 µM (69.66 ± 0.62% vs. 46.44 ± 4.47% in CTRL), accompanied by a reduction in the S and G2/M phase populations. At 10 µM, an increase in the S phase fraction was observed (11.3 ± 0.74%). Dapagliflozin at 20 µM resulted in a marked increase in G0/G1 phase cells (70.88 ± 1.34%) and a concomitant decrease in S and G2/M fractions. Empagliflozin promoted G0/G1 phase arrest at both tested concentrations (10 µM: 61.61 ± 1.98%; 20 µM: 68.76 ± 1.37%), reduced the S phase population at 20 µM, and decreased the G2/M fraction at both concentrations. A small proportion of events with DNA content >4N was observed under selected treatment conditions. However, these events were not interpreted further because doublet-discrimination gating and dedicated DNA-content modeling were not performed (Figure 3).

**Figure 4:**
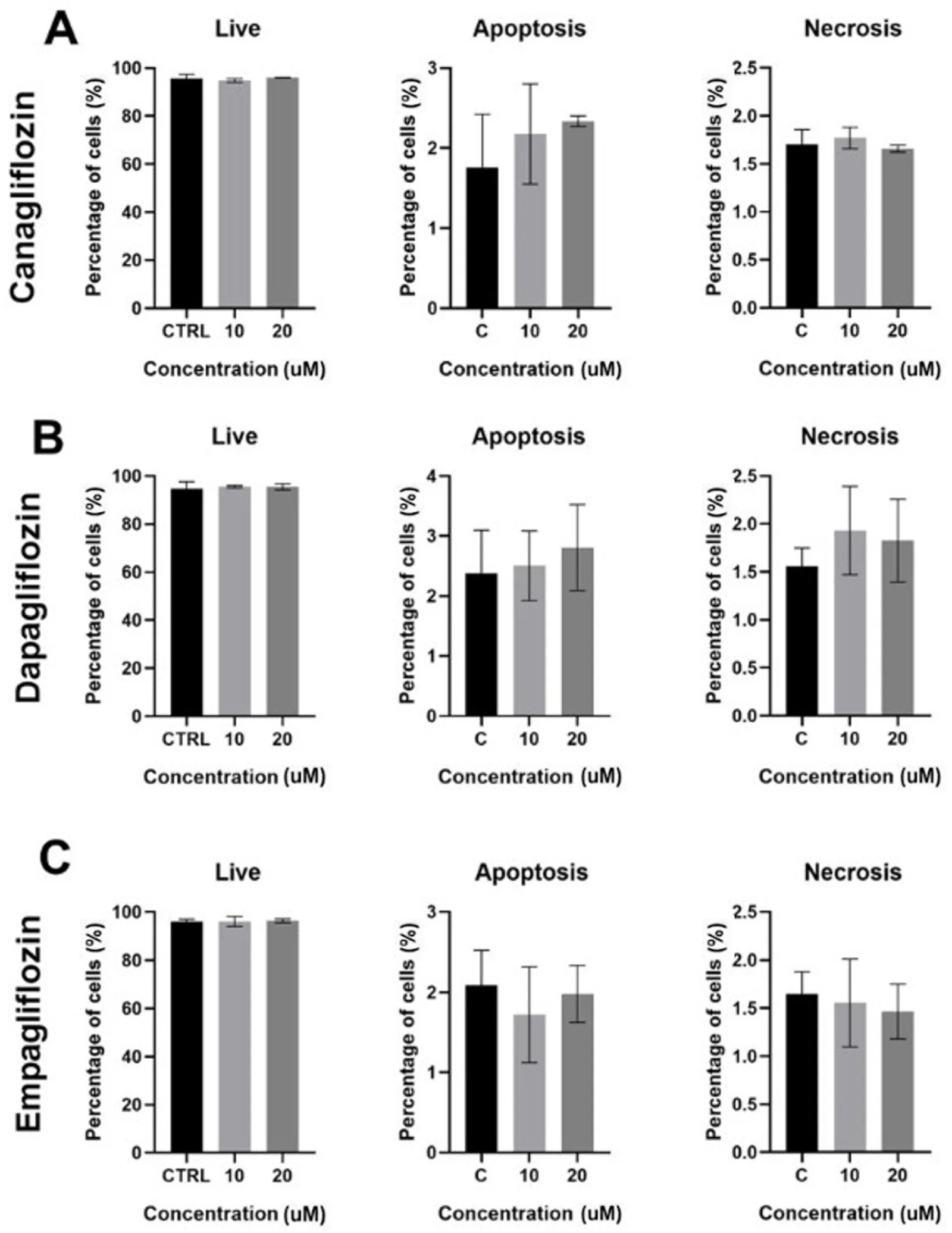
Analysis of cell death parameters in T24 cells after treatment with SGLT2 inhibitors. Analysis of live, apoptotic, and necrotic T24 cells following treatment with 10 and 20 µM of canagliflozin (**A**), dapagliflozin (**B**), and empagliflozin (**C**) for 24 h. Cell populations were determined by Annexin V/PI staining assay and quantified by flow cytometry. Data represent mean ± SD from 3 independent experiments (n = 3). Statistical analysis was performed using ordinary one-way ANOVA with Tukey’s multiple comparisons test. *p* < 0.05 vs. CTRL (control cells), SGLT2—sodium-glucose cotransporter 2.

Analysis of cell death parameters revealed that treatment with SGLT2 inhibitors did not markedly affect the overall proportion of live T24 cells (Figure 4). Following canagliflozin exposure, the percentage of live cells remained at 94.83 ± 0.7% and 96.05 ± 0.06% for 10 and 20 µM, respectively, compared with 95.64 ± 1.63% in CTRL. The proportion of apoptotic cells was 2.18 ± 0.63% (10 µM) and 2.34 ± 0.07% (20 µM), while necrotic cells accounted for 1.77 ± 0.11% and 1.66 ± 0.4%, respectively.

Similarly, dapagliflozin treatment maintained a high percentage of viable cells (10 µM: 95.67 ± 0.46%; 20 µM: 95.51 ± 1.25% vs. 94.67 ± 2.95% in CTRL). The fraction of apoptotic cells was 2.50 ± 0.58% and 2.81 ± 0.72% at 10 and 20 µM, respectively, whereas necrotic cells constituted 1.93 ± 0.46% and 1.82 ± 0.43%.

Empagliflozin exposure also did not substantially reduce the proportion of live cells (10 µM: 96.02 ± 2.08%; 20 µM: 96.37 ± 0.76% vs. 95.98 ± 2.1% in CTRL). The percentage of apoptotic cells reached 1.72 ± 0.6% and 1.98 ± 0.36% at 10 and 20 µM, respectively, while necrotic cells accounted for 1.56 ± 0.5% and 1.47 ± 0.29%. No statistically significant differences were observed unless otherwise indicated. These findings indicate that the reduction in MTT metabolic activity is unlikely to result from extensive apoptosis or necrosis after 24 h of treatment.

### 3.3. Flozins Modulate SGLT2 Protein Levels in T24 Cells

**Figure 5:**
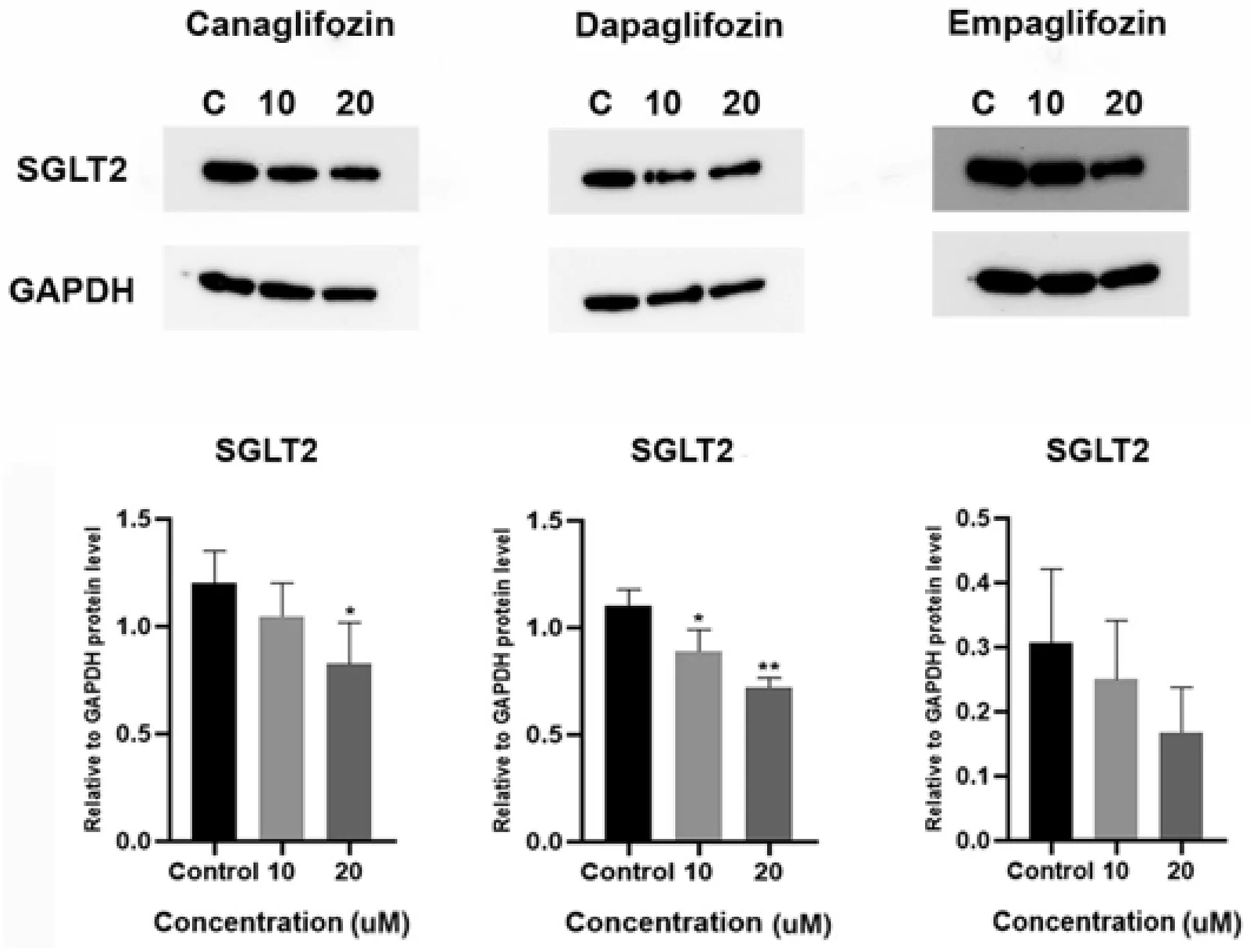
Modulation of SGLT2 protein levels in T24 cells following treatment with canagliflozin, dapagliflozin, and empagliflozin. Representative Western blots and densitometric analysis normalized to GAPDH are shown. Data are presented as mean ± SD from 3 independent experiments (n = 3). Statistically significant differences are indicated as * *p* < 0.05, ** *p* < 0.01 (ordinary one-way ANOVA with Tukey’s multiple comparisons test). CTRL—control cells, C—canagliflozin, D—dapagliflozin, E—empagliflozin SGLT2—sodium-glucose cotransporter 2, GAPDH—glyceraldehyde 3-phosphate dehydrogenase.

Western blot analysis revealed detectable SGLT2 protein expression in T24 cells (Figure 5). Treatment with SGLT2 inhibitors was associated with reduced SGLT2 protein expression, although the magnitude of the response differed between compounds.

Canagliflozin significantly reduced SGLT2 expression at 20 µM, while dapagliflozin induced a significant decrease at both 10 µM and 20 µM. Although empagliflozin treatment resulted in a downward trend in SGLT2 protein levels, these changes did not reach statistical significance.

### 3.4. SGLT2 Inhibitors Alter Migration Behavior of T24 Cells

**Figure 6:**
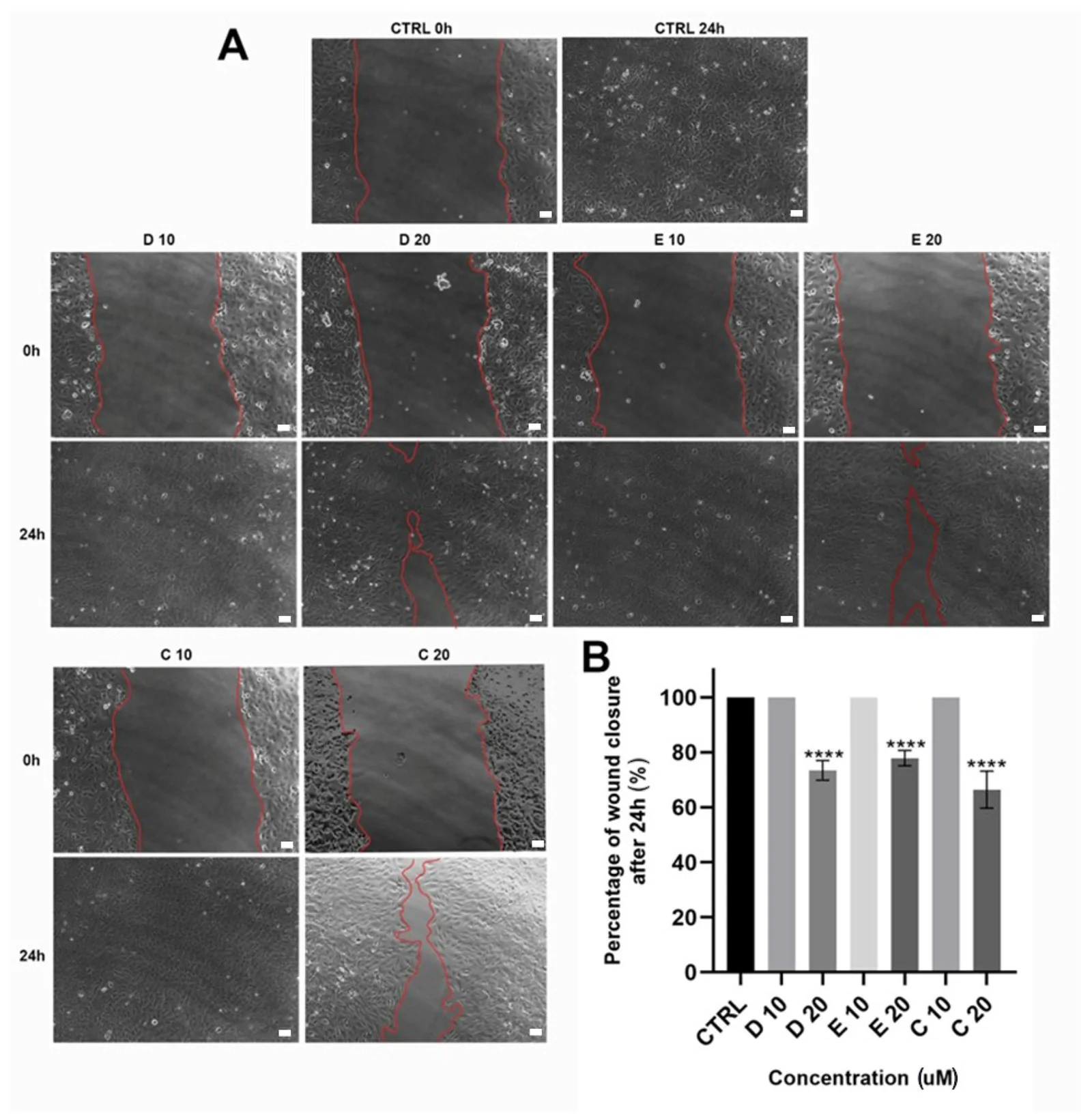
Effects of SGLT2 inhibitors on collective wound closure of T24 bladder cancer cells. Wound healing assay performed to evaluate collective wound closure of T24 cells after 24 h treatment with 10 and 20 µM of SGLT2 inhibitors. Wound closure was quantified and expressed as percentage of the initial wound area (CTRL = 100%). (**A**) Representative images of T24 cells at specific time points after scratching (0 and 24 h). Scale bar = 50 µm. (**B**) Statistical analysis of mean results for percentage wound surface healing. A closed wound was considered 100%. Results are presented as mean with standard deviation. Statistically significant differences are indicated as **** *p* < 0.0001 (ordinary one-way ANOVA with Dunnett’s multiple comparisons test). CTRL—control cells, C—canagliflozin, D—dapagliflozin, E—empagliflozin, SGLT2—sodium-glucose cotransporter 2.

**Figure 7:**
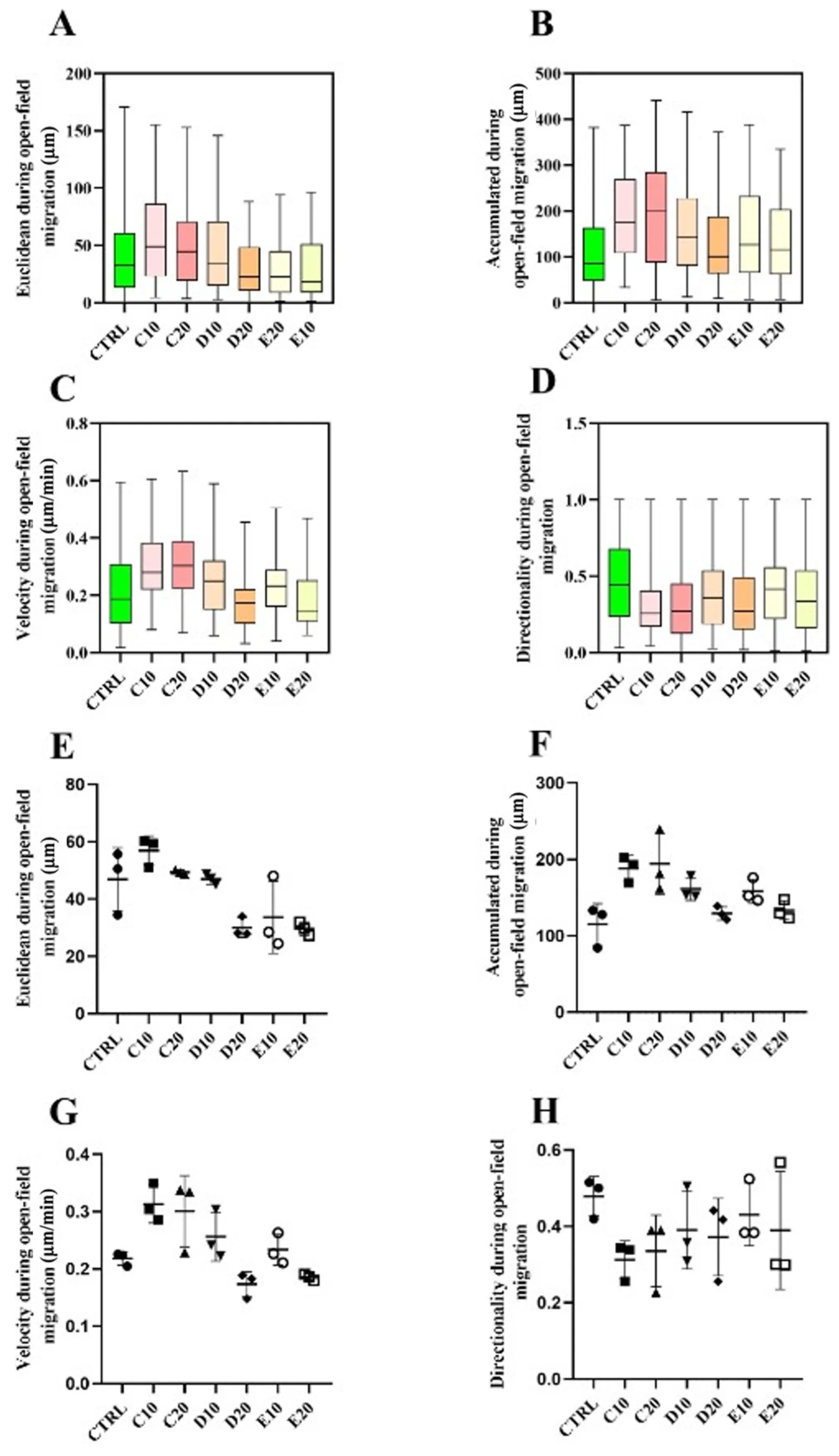
Effects of SGLT2 inhibitors on open-field migration parameters in T24 bladder cancer cells. (**A**), accumulated distance (µm) (**B**), velocity (µm/min) (**C**) and directionality (**D**). Individual cell measurements are presented to illustrate the distribution and heterogeneity of migration parameters and were not treated as independent biological replicates for inferential statistical analysis. Directionality was calculated as the ratio of Euclidean distance to accumulated distance, with higher values indicating more directional cell migration. Data are presented as box-and-whisker plots, where the center line represents the median, boxes indicate the interquartile range (25th–75th percentile), and whiskers denote the minimum and maximum values. (**E**–**H**) Biological replicate-level analysis of Euclidean distance (µm) (**E**), accumulated distance (µm) (**F**), velocity (µm/min) (**G**) and directionality (**H**). Individual cell measurements were averaged within each of three independent biological experiments for each experimental condition, and experiment-level means are shown as individual points (n = 3 biological replicates; mean ± SD). Replicate-level data were analyzed using the Friedman test for matched observations, followed, where appropriate, by Dunn’s multiple-comparisons test against CTRL. Significant overall effects among experimental conditions were observed for accumulated distance (*p* = 0.0110), migration velocity (*p* = 0.0162), and Euclidean distance (*p* = 0.0203), whereas no significant overall difference was detected for directionality (*p* = 0.2590). None of the individual treatment conditions differed significantly from CTRL after correction for multiple comparisons. CTRL—control; C—canagliflozin; D—dapagliflozin; E—empagliflozin, SGLT2—sodium-glucose cotransporter 2.

The effect of SGLT2 inhibitors on T24 cell migration was assessed using wound healing and open-field migration assays (Figure 6 and Figure 7). After 24 h, control cells exhibited complete wound closure. Treatment with canagliflozin, dapagliflozin, and empagliflozin reduced wound closure in a concentration-dependent manner, although the magnitude of the response differed between compounds. Dapagliflozin treatment resulted in wound closure of 100% at 10 µM and 73.38 ± 3.61% at 20 µM, with a significant reduction observed at the higher concentration. Similarly, empagliflozin-treated cells demonstrated wound closure of 100% and 77.8 ± 2.79% at 10 and 20 µM, respectively. Canagliflozin also impaired wound closure at the 20 µM dose (66.36 ± 6.77%) (Figure 6A,B).

To further characterize cell motility, single-cell migration was performed using the OF assay. Cell-level distributions revealed considerable heterogeneity in Euclidean distance, accumulated distance, velocity and directionality across the experimental conditions (Figure 7A–D). Because individual cells tracked within the same experiment were not considered independent biological replicates, inferential statistical analysis was performed using experiment-level means from three independent biological experiments (Figure 7E–H). At the biological replicate level, the Friedman test indicated significant overall differences among experimental conditions for accumulated distance (*p* = 0.0110), migration velocity (*p* = 0.0162), and Euclidean distance (*p* = 0.0203), whereas no significant overall difference was detected for directionality (*p* = 0.2590). However, Dunn’s multiple-comparisons test did not identify any individual flozins treatment condition that differed significantly from CTRL after correction for multiple comparisons. Thus, although treatment-related variation in single-cell migration parameters was observed, the replicate-level analysis does not support statistically significant differences between individual treatment conditions and CTRL.

### 3.5. Bioinformatic Analysis of SLC5A2-Associated Interaction Networks and Functional Pathways

**Figure 8:**
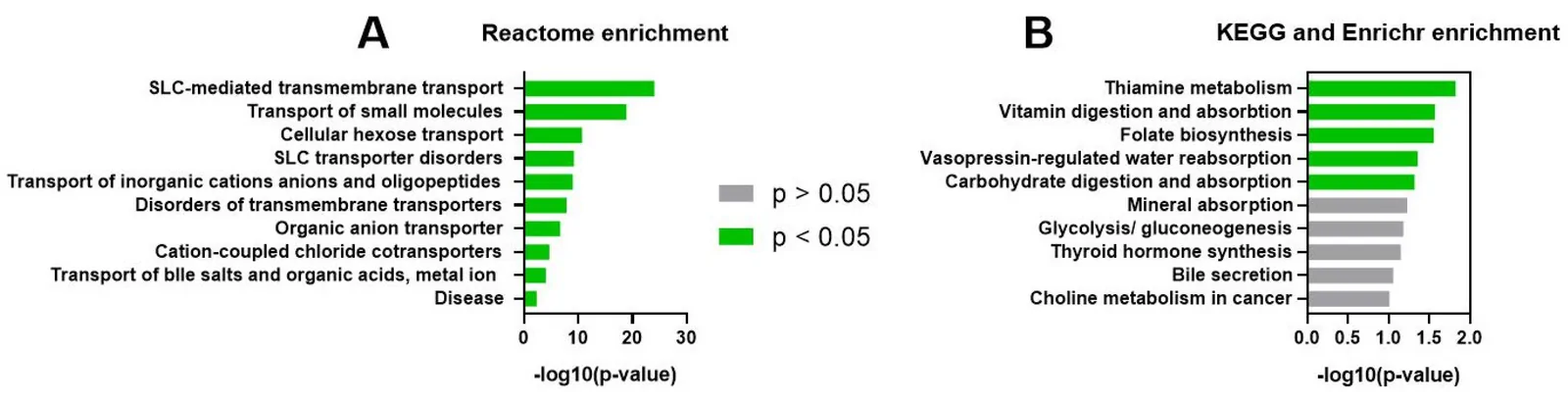
Functional enrichment analysis of genes associated with the *SLC5A2* interaction network using Enrichr, Reactome, and KEGG databases. (**A**) Reactome analysis identified enrichment of SLC-dependent transmembrane transport pathways. (**B**) KEGG and Enrichr analyses identified enrichment of pathways related to carbohydrate, vitamin, and mineral metabolism. These analyses are hypothesis-generating and do not demonstrate pathway activation or inhibition in T24.

To further characterize the biological context of the experimental findings, bioinformatic analyses were performed to investigate the interaction networks, functional pathways, and clinical relevance of *SLC5A2* (Figure 8, Figures S1 and S2 ).

#### 3.5.1. Gene and Protein Interaction Networks

To explore the functional context of *SLC5A2*, gene and protein interaction networks were examined using two independent platforms, GeneMANIA and STRING. Both approaches consistently showed that *SLC5A2* is functionally associated with other members of the SLC5 transporter family, including *SLC5A1*, *SLC5A3*, *SLC5A4*, *SLC5A5*, *SLC5A6*, *SLC5A7*, *SLC5A8*, and *SLC5A12* (Figures S1 and S2). The GeneMANIA network consisted predominantly of physical protein–protein interactions (~80%), together with co-expression and predicted functional associations. Gene ontology analysis indicated enrichment for sodium symporter activity and carbohydrate transmembrane transport [33,34].

#### 3.5.2. Pathway Enrichment Analysis

Functional enrichment analysis was performed using Enrichr based on Reactome and KEGG pathway databases to identify biological processes associated with the *SLC5A2* interaction network.

Reactome pathway analysis showed enrichment for processes related to SLC-dependent transmembrane transport, small molecule transport, and the transport of cations, anions, and inorganic amino acids, suggesting a role for membrane transport systems (Figure 8A) [35,36,37,38].

These findings were supported by KEGG and Enrichr analyses, which additionally highlighted pathways involved in carbohydrate digestion and absorption, vitamin digestion and absorption, mineral absorption, and glycolysis-related processes (Figure 8B) [35,36,37,39,40,41].

Collectively, the bioinformatic analyses identified functional associations of *SLC5A2* with transmembrane transport, carbohydrate metabolism, and other members of the SLC5 transporter family, providing biological context for the experimental observations and generating hypotheses for future mechanistic studies.

#### 3.5.3. Genomic, Clinical and Regulatory Context of *SLC5A2* in Bladder Cancer

**Figure 9:**
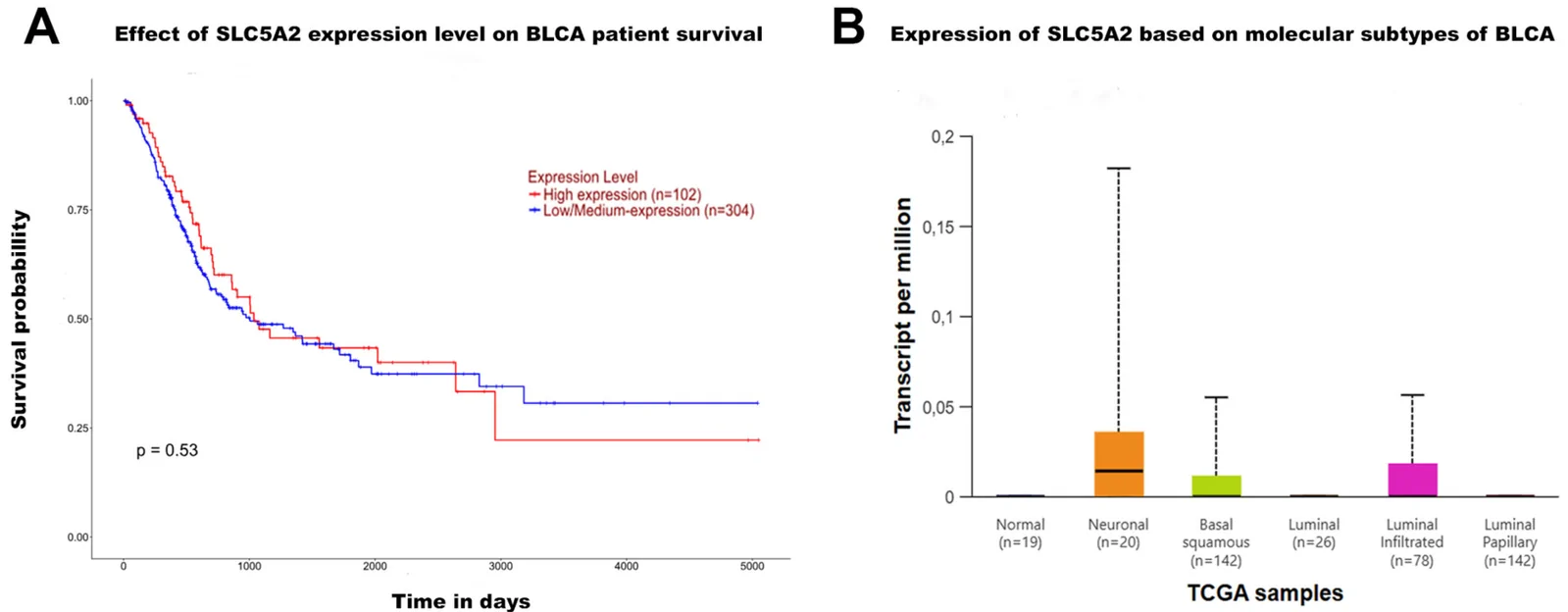
Clinical relevance of *SLC5A2* expression in bladder cancer based on TCGA data (UALCAN platform). (**A**) Kaplan–Meier survival analysis showing no significant difference in overall survival (OS) between high and low/medium *SLC5A2* expression groups (*p* = 0.53). (**B**) *SLC5A2* expression levels across molecular subtypes of bladder cancer, demonstrating variability in transcript abundance between subtypes. BLCA—bladder urothelial carcinoma; OS—overall survival; TCGA—The Cancer Genome Atlas [26,27].

Analysis of the TCGA Bladder Urothelial Carcinoma dataset in cBioPortal showed that genomic alterations in *SLC5A2* occurred in approximately 2% of cases, mainly gene amplifications or missense mutations. The majority of samples showed no detectable alterations, indicating that genomic alterations of *SLC5A2* occur infrequently in bladder cancer [28,29,30]. Survival analysis based on TCGA BLCA data showed no significant association between *SLC5A2* expression levels and overall survival (*p* = 0.53), with comparable outcomes between patients with high and low/medium expression (Figure 9A) [26,27].

Analysis of *SLC5A2* expression across molecular subtypes showed variability in transcript levels, with higher expression observed in selected subtypes compared with normal tissue, indicating variability in *SLC5A2* expression among molecular subtypes (Figure 9B).

Potential miRNA regulators of *SLC5A2* were identified using the miRDB database. Four candidate microRNAs were predicted, including hsa-miR-939-5p, hsa-miR-5002-3p, hsa-miR-1343-5p, and hsa-miR-4316, with miR-939-5p and miR-5002-3p showing the highest prediction scores. These analyses identified several candidate miRNAs for future investigation as potential regulators of *SLC5A2* expression.

Overall, the bioinformatic analyses provided complementary information regarding the molecular context, clinical characteristics, and functional associations of *SLC5A2*, supporting its further investigation in bladder cancer.

## 4. Discussion

SGLT2 inhibitors are widely used in clinical practice for the treatment of diabetes, and emerging evidence suggests that they may also exert effects in cancer models. However, their potential role in bladder cancer biology remains insufficiently understood. In this study, we investigated the effects of canagliflozin, dapagliflozin, and empagliflozin on T24 bladder cancer cells, focusing on metabolic activity, cell cycle distribution, and migratory capacity, as a first step toward characterizing their biological activity in this model.

Our results show that treatment with canagliflozin, dapagliflozin, and empagliflozin reduces the metabolic activity of the T24 bladder cancer cell line in a concentration-dependent manner. Treatment with canagliflozin, dapagliflozin, and empagliflozin at concentrations ranging from 10 µM to 30 µM decreased T24 cell metabolic activity, with all three compounds producing a broadly comparable reduction across the tested concentration range. Because the MTT assay reflects mitochondrial metabolic activity rather than cell number directly, and because canagliflozin is known to directly inhibit mitochondrial complex I independently of SGLT2, part of the observed reduction in MTT signal, particularly for canagliflozin, cannot be firmly attributed to reduced cell proliferation without orthogonal assays such as direct cell counting or EdU incorporation. Nonetheless, the observed reduction in metabolic activity is broadly consistent with previous reports describing antiproliferative effects of multiple SGLT2 inhibitors across a range of cancer cell lines, including Caco-2, A-549, Du-145, and Panc-1, although the relative potency of individual compounds has been shown to vary between cell types and experimental conditions [42]. Differences in the biological activity of individual flozins have been attributed in part to the lower SGLT2 selectivity of canagliflozin and its reported off-target effects, including inhibition of mitochondrial complex I, glutamate dehydrogenase, and AMP-activated protein kinase (AMPK) activation [25,43,44,45]. In the present study, nonlinear concentration–response analysis yielded similar estimated IC_50_ values for canagliflozin (31.30 µM), dapagliflozin (28.89 µM) and empagliflozin (30.78 µM), indicating broadly comparable effects on MTT-derived metabolic activity within the tested concentration range. Since empagliflozin has no reported direct mitochondrial complex I activity, the observed reduction in MTT-based viability following empagliflozin treatment in T24 cells is consistent with recent literature describing dose-dependent anticancer effects across Caco-2, A2780, and LNCaP cell lines, suggesting that even highly selective SGLT2 inhibitors may retain meaningful anticancer activity [46]. Overall, these findings provide preliminary evidence of flozin-induced reductions in MTT-derived metabolic activity, warranting further investigation of the underlying cellular processes using orthogonal proliferation assays. The present data do not support a clear potency ranking among the three compounds. Importantly, the estimated IC_50_ values were close to the upper boundary of the tested concentration range and should therefore be interpreted cautiously.

Western blot analysis confirmed the presence of SGLT2 protein in T24 bladder cancer cells, while immunofluorescence analysis demonstrated a diffuse intracellular distribution of the SGLT2 signal, with local enrichment in peripheral cellular regions adjacent to cortical F-actin structures. This distribution differs from the well-established luminal membrane localization of SGLT2 in the renal proximal tubule [10]. Similar intracellular patterns of SGLT2 immunoreactivity have also been reported in several tumor models, suggesting that the subcellular distribution of SGLT2 may differ between normal renal tissue and cancer cells [15]. This intracellular, diffuse distribution differs from the classical membrane localization of SGLT2 and has not been independently confirmed using complementary techniques such as co-staining with membrane and organelle markers, cell-surface biotinylation, or subcellular fractionation. Such validation was not performed in the present study and represents a limitation of the immunofluorescence data presented here. SGLT2 expression has been reported to be absent or very low in normal urothelial tissue, whereas its presence has been described in several cancer models [15,47,48]. Given the increased glucose demand and metabolic reprogramming characteristic of many malignant cells, including the Warburg phenotype, SGLT2 expression in tumor cells may represent part of a broader metabolic adaptation. However, this possibility remains to be experimentally established in bladder cancer. Dynamic regulation of SGLT2 trafficking represents one possible explanation, although the present study provides no direct evidence for this mechanism. SGLT2-mediated glucose uptake has been described in several other tumor types, including glioblastoma, pancreatic, and prostate cancers, suggesting that it may reflect a broader pattern of metabolic adaptation beyond renal physiology [49]. This is further supported by previous work showing that canagliflozin reduced glucose uptake, lactate production, and intracellular ATP selectively in SGLT2-expressing Huh7 and HepG2 liver cancer cells, with no such effect in SGLT2-null HLE cells, which may lend indirect support to a functional role for SGLT2 in T24 cells [17]. These observations provide a rationale for investigating whether SGLT2 contributes to glucose handling in bladder cancer cells; however, transporter activity and glucose uptake were not assessed in the present study. An additional finding of the present study was the reduction in SGLT2 protein levels following flozin treatment. The magnitude of this response differed between compounds, with the most pronounced changes observed following dapagliflozin treatment at 10 and 20 µM and canagliflozin treatment at 20 µM. These findings raise the possibility that SGLT2 inhibitors may influence not only transporter activity but also SGLT2 protein abundance. Alterations in protein abundance may potentially involve changes in intracellular trafficking or protein turnover, as described for other membrane-targeted proteins following pharmacological treatment, including erlotinib [50]. However, the mechanism underlying the reduction in SGLT2 protein levels observed in the present study was not investigated. Altered protein turnover, trafficking, transcriptional regulation, or other post-translational processes therefore remain possible explanations that require dedicated experimental validation. It should also be noted that the present study demonstrates SGLT2 expression in T24 cells and shows that flozin treatment is associated with altered cellular phenotypes but does not establish that these phenotypic effects are causally mediated by SGLT2 inhibition itself. *SLC5A2* knockdown, knockout, or rescue experiments would be required to confirm target specificity.

Flow cytometry analysis using Annexin V/PI staining showed that exposure to SGLT2 inhibitors at 10 µM and 20 µM did not increase apoptosis or necrosis within the 24 h timeframe, with the live cell population remaining near 95%. These findings indicate that the reduction in metabolic activity observed in the MTT assay was not accompanied by a pronounced increase in cell death under the experimental conditions used. In contrast, cell cycle analysis demonstrated an accumulation of T24 cells in the G0/G1 phase, accompanied by a reduction in the S and G2/M populations, supporting a predominantly cytostatic response associated with altered cell cycle progression. This pattern of cell cycle arrest is consistent with previous observations in other cancer models, where SGLT2 inhibition has been associated with similar effects [51]. The molecular mechanisms responsible for the observed G0/G1 accumulation in T24 cells were not investigated in the present study. However, previous studies provide several possible mechanisms that may be relevant. In thyroid cancer cells, canagliflozin was reported to inhibit the G1/S transition through changes in cyclins D1, D3, E1, E2, and E2F1, together with activation of the ATM/CHK2 pathway and increased γ-H2AX expression [52]. These findings suggest that metabolic or energetic stress associated with SGLT2 inhibition may, in some cellular contexts, interact with DNA damage and cell cycle checkpoint pathways. Whether similar mechanisms contribute to the response of T24 cells remains to be established. Some treatment- and concentration-dependent differences in cell cycle distribution were also observed. Canagliflozin at 10 µM was associated with an increased proportion of cells in S phase, which may be compatible with altered cell cycle progression or replication stress. Dapagliflozin at 10 µM was associated with an increased proportion of cells with DNA content above 4N. Although this population may reflect polyploidization or other biological changes, technical factors such as cell aggregation or doublets cannot be excluded based on the present analysis. Therefore, the biological significance of the >4N population should be interpreted cautiously. No doublet-discrimination gating (e.g., FSC-H versus FSC-A) or DNA content modeling (e.g., Watson or Dean–Jett–Fox models) was applied in the present analysis, so this finding cannot be confidently interpreted and should be treated as exploratory only. Previous observations that dapagliflozin can affect cell adhesion through DDR1 cleavage and ADAM10 activity in other cellular models provide an additional potential context for altered cell cycle behavior; however, whether adhesion-dependent signaling contributes to the observed changes in T24 cells was not investigated [53]. Overall, the combined Annexin V/PI and cell cycle data support the interpretation that, under the conditions tested, flozin treatment is associated primarily with altered cell cycle progression rather than a pronounced induction of apoptosis or necrosis. Further studies assessing G1/S regulatory proteins and checkpoint signaling will be required to determine the molecular mechanisms underlying this response.

Beyond their effect on cell cycle progression, SGLT2 inhibitors also altered collective wound closure in T24 bladder cancer cells, with more pronounced effects generally observed at higher concentrations. Open-field analysis additionally revealed heterogeneous patterns of single-cell motility across treatment conditions. Although the cell-level distributions suggested compound- and concentration-dependent differences in accumulated distance, Euclidean displacement, velocity and directionality, these individual cell measurements were not considered independent biological replicates. When the data were reanalyzed at the level of three independent biological experiments, significant overall effects were detected for accumulated distance, velocity, and Euclidean distance, but none of the individual treatment conditions differed significantly from CTRL after correction for multiple comparisons. Therefore, these patterns should be interpreted as treatment-associated variation in migratory behavior rather than evidence of a statistically confirmed increase or decrease in motility for a specific flozin. Because the wound healing assay cannot clearly distinguish inhibition of migration from inhibition of cell proliferation, and because the same treatments induced G0/G1 cell cycle accumulation, the reduced wound closure reported here may partly reflect decreased proliferation rather than impaired migration alone. Additional experiments that control for proliferation, such as pretreatment with a proliferation inhibitor (e.g., mitomycin C), would be required to disentangle these contributions.

The apparent discrepancy between impaired collective wound closure and the heterogeneous patterns observed in single-cell motility is particularly relevant for interpreting the effects of flozins on T24 cells. Such changes, if confirmed in larger replicate-level datasets, could potentially influence the efficiency of coordinated population-level movement. At the same time, the G0/G1 accumulation observed in the cell cycle analysis may further limit the contribution of cell proliferation to wound closure. Thus, the present findings support an interpretation in which flozin treatment affects the coordination and efficiency of collective cell behavior rather than simply inhibiting the ability of individual cells to move.

A particularly interesting pattern was observed for canagliflozin. At the descriptive single-cell level, canagliflozin-treated cells tended to show higher migration velocity and accumulated distance than CTRL, whereas dapagliflozin- and empagliflozin-treated cells showed different patterns of migratory behavior. Importantly, these apparent treatment-specific differences were not confirmed as significant in the replicate-level post hoc comparison and should therefore be interpreted cautiously. Nevertheless, the divergent pattern observed for canagliflozin is noteworthy because it may reflect compound-specific, SGLT1-independent activity. Canagliflozin has well-documented off-target activities not shared by the other two flozins, including direct inhibition of mitochondrial complex I, inhibition of glutamate dehydrogenase, and activation of AMPK independent of SGLT2 blockade [43,44,45]. Any of these effects could plausibly alter cytoskeletal dynamics or energy-dependent motility machinery through SGLT2-independent pathways. We therefore cannot exclude that the apparent pro-migratory pattern observed for canagliflozin at the descriptive single-cell level may reflect such off-target activity rather than SGLT2 inhibition itself. This discrepancy shows that, while all three compounds reduced viability and induced G0/G1 arrest to a broadly comparable extent, their effects on single-cell motility were not uniform, and a common SGLT2-dependent mechanism cannot be assumed to underlie all of the observed phenotypes. Distinguishing on-target from off-target contributions to migration, for example through *SLC5A2* knockdown or a more SGLT2-selective inhibitor panel, will be an important direction for future work.

Previous studies have reported that flozins can interfere with signaling pathways involved in cell growth, metabolism, and cellular organization, including mechanistic target of rapamycin (mTOR)- and epidermal growth factor receptor (EGFR)-related pathways [54]. These pathways may provide a potential molecular context for the altered migratory behavior observed in the present study; however, their involvement in the response of T24 cells to SGLT2 inhibitors was not directly examined. Further studies will therefore be required to determine whether changes in these signaling pathways contribute to the altered migration dynamics observed following flozin treatment.

Bioinformatic analysis showed that *SLC5A2* is functionally associated with other members of the SLC transporter family within a functional network involved in molecule uptake and cellular transport. These findings provide additional biological context for the experimental observations and support the potential involvement of transport- and metabolism-related processes in the response of T24 cells to flozin treatment. However, the present analyses do not establish whether these pathways directly mediate the observed cellular effects. It is worth stressing that associations from STRING, GeneMANIA, Enrichr, KEGG, Reactome, TCGA, and miRDB do not show that these pathways are actually activated or inhibited by SGLT2 inhibitors in T24 cells. They are hypothesis-generating, not direct evidence for the proposed mechanism, and the key pathways identified here would need experimental validation before any causal role can be established. Given the increased glucose demand and metabolic reprogramming characteristic of cancer cells, altered nutrient transport represents a plausible area for further investigation, but glucose uptake and metabolic parameters were not directly assessed in the present study. TCGA-based analysis showed no significant association between *SLC5A2* expression and overall survival in patients with bladder cancer. This finding does not exclude a potential biological role of SGLT2 in tumor cells but indicates that *SLC5A2* expression alone should not be considered an established prognostic marker in bladder cancer. The functional effects observed in the present in vitro model, together with the absence of a significant survival association, suggest that the biological relevance of SGLT2 in bladder cancer may not be adequately captured by survival analysis alone. Further studies integrating *SLC5A2* expression with functional and metabolic parameters will be required to clarify its relevance in bladder cancer.

The safety profile of SGLT2 inhibitors in relation to bladder cancer has been debated, particularly following earlier reports suggesting a potential increased risk associated with glucosuria [55,56]. However, a series of recent large-scale population analyses have consistently shown that gliflozin therapy does not increase bladder cancer incidence or metastasis risk [57,58,59,60,61]. These clinical observations provide an important context for investigating the biological effects of flozins in bladder cancer models. Nevertheless, the present in vitro findings should not be interpreted as evidence of a clinical antitumor effect or as evidence that the anticancer activity of flozins offsets any potential cancer-related risks. Broader oncology studies have proposed several mechanisms through which SGLT2 inhibitors may affect tumor cells, including AMPK activation, alterations in mitochondrial function, and changes in tumor-associated metabolic signaling [25,51,58,62,63]. However, our study was not designed to test these specific pathways, and whether similar processes contribute to the effects we observed in T24 cells remains an open question. Taken together, these observations provide a rationale for further investigation of the biological effects of SGLT2 inhibitors in bladder cancer, while their clinical relevance remains to be established.

An important limitation of the present study is the concentration range used for the in vitro experiments, which exceeds typical plasma concentrations achieved with standard clinical dosing, particularly for empagliflozin and dapagliflozin. Although similar concentrations have been used in previous in vitro studies investigating the biological effects of SGLT2 inhibitors, in vitro exposure cannot be directly equated with clinically achievable systemic concentrations [64,65,66]. Tissue distribution, protein binding, local drug exposure, and differences in intracellular drug accumulation may influence the effective concentration within a tumor; however, these parameters were not evaluated in the present study. Therefore, the observed effects should be regarded as evidence of cellular responsiveness under experimental conditions rather than as an indication of clinical efficacy. The specific pathways underlying the antiproliferative effects of SGLT2 inhibitors in bladder cancer cells were not addressed in the present study. However, findings from breast cancer, glioblastoma, and cervical carcinoma point to metabolic regulation, particularly AMPK and its downstream interaction with mTOR signaling, as a potentially relevant pathway. In glioblastoma models, canagliflozin has been shown to activate AMPK and suppress mTOR signaling, leading to decreased cell proliferation [67]. Similar findings have been reported in breast cancer, where AMPK activation was associated with inhibition of mTOR signaling [51], and in cervical cancer, where AMPK activation led to suppression of downstream targets such as SHH [68]. Together, these reports suggest that AMPK may act as a regulator of tumor cell proliferation across several cancer types. The G0/G1 cell cycle arrest observed in T24 cells following SGLT2 inhibition is consistent with this broader pattern, raising the possibility that related processes could contribute to the effects observed here. Future studies directly assessing AMPK/mTOR signaling and other cell cycle regulators in bladder cancer models will be needed to clarify the mechanisms underlying these effects.

## 5. Limitations

Several limitations of this study should be considered when interpreting the findings. First, the experiments were performed using a single bladder cancer cell line (T24), without inclusion of a normal urothelial comparator. Additional bladder cancer models, together with non-transformed urothelial cells, will be important to determine the reproducibility and selectivity of the observed effects. Although previous studies have reported absent or very low SGLT2 expression in normal urothelium [15,47,48], this could not be directly evaluated in the present experimental model. Second, the study was designed primarily as a phenotypic characterization rather than a mechanistic investigation. We did not directly assess AMPK/mTOR signaling, G1/S cell-cycle regulatory proteins, glucose uptake, glycolysis, or mitochondrial function. In addition, cell viability was assessed using the MTT assay, which reflects mitochondrial metabolic activity rather than cell number directly. Because canagliflozin is a direct inhibitor of mitochondrial complex I, part of the observed reduction in MTT signal cannot be firmly distinguished from reduced cell proliferation without orthogonal assays such as direct cell counting, EdU incorporation, or Ki-67 staining, none of which were performed in the present study. These parameters may help clarify the mechanisms underlying the observed cell viability and represent important directions for future studies. Similarly, the functional assays were performed over a 24 h period. Longer time-course experiments, including 48 and 72 h, would help determine whether the observed effects persist, increase, or change over time. For the open-field migration assay, inferential statistical analysis was performed at the level of three independent biological experiments rather than individual tracked cells. Although this approach avoids pseudoreplication, the small number of biological replicates limits statistical power, and the observed treatment-associated patterns in single-cell motility therefore require confirmation in larger independent datasets. An additional exploratory finding was the reduction in SGLT2 protein levels following flozin treatment. Although this observation may be biologically relevant, the present study did not determine whether the altered protein abundance results from changes in protein turnover, intracellular trafficking, transcriptional regulation, or other regulatory mechanisms. This finding should therefore be considered preliminary and warrants dedicated mechanistic investigation. In addition, the present study did not include genetic manipulation of *SLC5A2*. Therefore, the observed phenotypes cannot be considered definitively SGLT2-dependent. *SLC5A2* knockdown/knockout and rescue experiments will be required to establish target specificity. In turn, measurement of *SLC5A2* mRNA will be required to determine whether the observed reduction in SGLT2 protein abundance includes a transcriptional component, while dedicated protein-stability and trafficking experiments would be necessary to evaluate post-translational mechanisms.

Finally, the concentrations of SGLT2 inhibitors used in the present study were higher than typical plasma concentrations achieved with standard clinical dosing. Although concentrations within this range have been used in previous in vitro studies, in vitro exposure cannot be directly equated with clinically achievable systemic exposure. Therefore, the biological effects observed here should be interpreted within the experimental context, and their clinical relevance remains to be established. Despite these limitations, the present study provides an initial phenotypic characterization of the effects of three clinically used SGLT2 inhibitors on bladder cancer cells, including metabolic activity, cell cycle progression, collective wound closure, and single-cell migration behavior. The integration of functional experiments with exploratory bioinformatic analyses of *SLC5A2* provides a framework for future studies aimed at determining whether the observed effects are mediated through SGLT2-dependent mechanisms or broader metabolic and transport-related processes.

## 6. Conclusions

This study provides a preliminary characterization of the effects of canagliflozin, dapagliflozin, and empagliflozin on T24 bladder cancer cells. All three compounds reduced MTT-derived metabolic activity and were associated with G0/G1 cell cycle accumulation, without a significant increase in apoptosis or necrosis at 24 h. Flozin treatment also impaired collective wound closure and altered single-cell migration dynamics. SGLT2 protein was detected in T24 cells and its abundance changed following flozin treatment. However, the present study does not establish that the observed cellular effects are causally mediated by SGLT2. These findings provide a basis for further target-validation and mechanistic studies of flozin-responsive pathways in bladder cancer.

## Data Availability

The original contributions presented in this study are included in the article/Supplementary Materials. Further inquiries can be directed to the corresponding author.

## Supplementary Materials

The following supporting information can be downloaded at https://www.mdpi.com/article/10.3390/biomedicines14092037/s1, Figure S1: Gene interaction network of SLC5A2. Figure S2: Gene Protein–protein interaction network of SLC5 family transporters.
