# Supplementary material for: Preliminary Evaluation of SGLT2 Inhibitors in Human Bladder Cancer T24 Cells
Source: Biomedicines. 2026 Sep 10;14(9):2037. doi: 10.3390/biomedicines14092037 (PMC13604264; doi:10.3390/biomedicines14092037)
Supplement: Supplementary file 1 [file biomedicines-14-02037-s001.zip › biomedicines-4526322-supplementary.pdf]

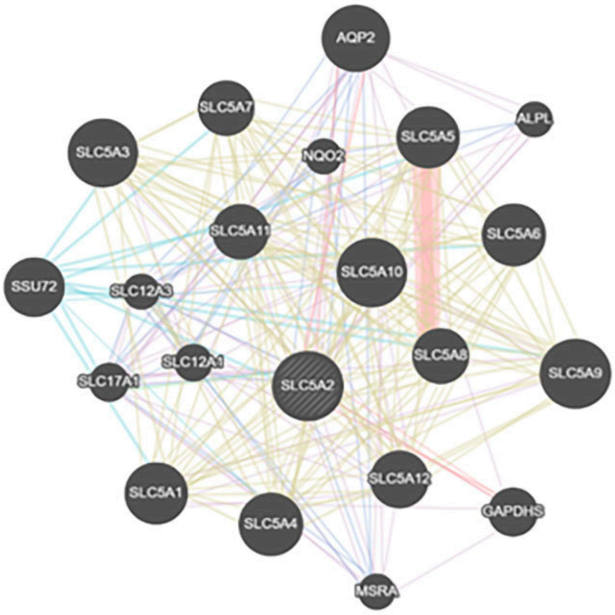

Figure S1: **Gene interaction network of SLC5A2** generated using the GeneMANIA platform showing functional associations with members of the SLC transporter family.

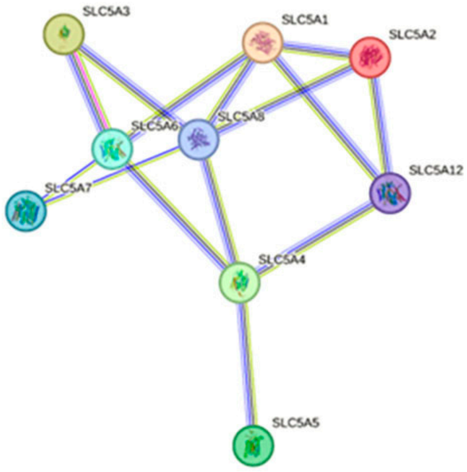

Figure S2: **Gene Protein–protein interaction network of SLC5 family transporters** generated using the STRING database.
